# Ten years of the Brazilian Researchers’ Meeting on Alzheimer’s Disease and Related Disorders

**DOI:** 10.1590/S1980-57642008DN10400001

**Published:** 2007

**Authors:** Ricardo Nitrini

**Affiliations:** Editor-in-Chief

In 1997, the First Brazilian Researchers’ Meeting on Alzheimer’s Disease and Related Disorders was held in São Paulo, where this year marks the VI meeting of the group. These meetings have been sponsored by the Cognitive Neurology and Ageing Department (CNAD) of the Brazilian Academy of Neurology, and from the outset the following objectives have been pursued: to promote a gathering of clinical and basic researchers to increase knowledge of ongoing research in Brazil, to stimulate the creation of new research groups and of cooperative multidisciplinary studies. One peculiar feature of this meeting is that registration is only possible through the submission of an abstract.

The success of this project is owing to two main factors: the leadership of the CNAD, and the remarkable support of all research groups of the field in Brazil. In the First Meeting, 51 abstracts were presented from several Brazilian centers. In the meetings that have followed, the number of abstracts has progressively increased and now in 2007, in the forthcoming Meeting to be held in Ouro Preto, 210 abstracts shall be presented in platform or poster form.

The meetings have spawned several successful projects, most notably the norms for the diagnosis and treatment of Alzheimer’s disease in Brazil,^1-3^ and the glossary of Brazilian-Portuguese terms which are frequently used in neurosciences of cognition and behavior.^4^

The scientific output of Brazilian neuroscientists in the field of dementia has increased over the last ten years. From 1998 to 2002, 96 papers on the field were indexed in Medline, while from 2003 to 30^th^ November 2007, 221 papers had been indexed, representing a significant 2.3 fold increase. The Researchers’ Meeting on Alzheimer’s Disease and Related Disorders is certainly partially responsible for this growth.

Abstracts have been published in English and Portuguese and from the Second to the Fifth Meeting were published as supplements of Arquivos de Neuro-Psiquiatria, the official journal of the Brazilian Academy of Neurology. Dementia & Neuropsychologia, in its capacity as the official journal of the CNAD, has now the privilege of publishing the abstracts in a special supplement.
